# Multimedia design for learner interest and achievement: a visual guide to pharmacology

**DOI:** 10.1186/s12909-024-05077-y

**Published:** 2024-02-05

**Authors:** Tyler Bland, Meize Guo, Tonia A. Dousay

**Affiliations:** 1 WWAMI Medical Education Program, University of Idaho, Moscow, ID USA; 2 College of Education Kenneth C. Griffin Computer Science Education for All Initiative , University of Florida , Gainesville , FL USA; 3 School of Education, University of Alaska Anchorage, Anchorage, AK USA

**Keywords:** Multimedia learning, Design principles, Situational interest, Student motivation, Student achievement

## Abstract

**Background:**

Medical education increasingly relies on digital learning materials. Despite recognition by the Association of American Medical Colleges Institute for Improving Medical Education, medical education design often fails to consider quality multimedia design principles. Further, the AAMC-IIME issued a call to study the role of design principles in medical education. Thus, the current study investigated the cognitive and affective effects of redesigning *PowerPoint* slides used to support pharmacology content during the preclinical years of medical school training.

**Methods:**

Researchers implemented a quasi-experimental design, using traditionally-designed (original) slides with a Traditional group (*n* = 100) and slides redesigned to follow principles from the Cognitive Theory of Multimedia Learning with an Experimental group (*n* = 40). Participants in the Experimental group completed a post-survey incorporating the Situational Interest Survey for Multimedia to indicate motivational engagement with the media. Students in the Experimental group also responded to additional preference questions. Researchers analyzed survey responses and students’ scores in pharmacology-related coursework across the preclinical Foundations Phase of training to determine the impact on achievement and motivation.

**Results:**

Findings related to learner achievement showed a modest but significant increase in the Experimental group compared to the Traditional group in the Cardiac, Pulmonary, and Renal (CPR) educational block (105%, normalized to Traditional group, *p* = 0.013) and cumulative pharmacology grades (101%, normalized to Traditional group, *p* = 0.043). Additionally, participants in the Experimental group indicated a significantly higher average triggered situational interest in redesigned slides (M = 4.85, SD = 0.25) than the original slides (M = 3.23, SD = 1.40, t=-6.33, *p* < 0.001). Similarly, the interest rating of the redesigned slides (M = 4.87, SD = 0.24) was significantly greater than that of the original slides (M = 3.89, SD = 0.86, t=-6.824, *p* < 0.001). Moreover, results further indicated significant differences in the maintained-feeling and maintained-value constructs, and all participants in the Experimental group indicated a preference for the redesigned slides.

**Conclusions:**

The findings provide strong evidence in support of using the Cognitive Theory of Multimedia Learning design principles to intentionally design media in medical education. In addition to increased achievement scores, students in the Experimental group demonstrated significantly higher levels of situational interest in the redesigned slides, especially triggered interest and maintained-feeling interest. Medical education learning designers should seriously consider redesigning media to achieve similar results.

## Background

In an electronic age where the majority of medical students learn in a digital format, the design and approachability of multimedia curricula has a substantial impact on learners. Multimedia in the context of learning refers to using words, printed or spoken, in juxtaposition to images, static or dynamic, which includes everything from slide presentations to videos and animations [1]. A synthesis of research in the learning sciences and instructional design prescribe design principles guiding multimedia design and use depending upon the content and context that the media is presented. These design principles are especially important for learning experiences where the germane load will be high, as in the case with teaching complex concepts and systems commonly found in medical education [2]. In this case, using structural aids, such as flowcharts or diagrams, metaphors, prompts, and layered details, helps learners form connections between new and existing knowledge [3]. This research-based finding forms the foundation for the *Cognitive Theory of Multimedia Learning* (CTML), which establishes a clear relationship between cognitive benefits and using both words and images to support learning [4]. However, medical education does not often refer to established media design recommendations despite their value, and there are calls for evidence-based practice on applying educational theories in medical education design [5, 6]. The current study responds to this call to action, drawing upon cognitive psychology theories and practices.

When using media to support learning, instructors and learning designers must acknowledge learners’ cognitive and affective processes [7]. From the original work proposing *Information-Processing Theory* (IPT) through *Dual-Coding Theory* (DCT) to *Cognitive Load Theory* (CLT), the learning sciences stand on more than fifty years of confirmed studies understanding the basic actions, affordances, and limitations of using words, pictures, and audio to promote learning [8–11]. IPT confirms that humans receive information via a sensory registry and process this information in short-term memory before storing and retrieving the information in long-term memory. DCT extends IPT by specifying that we have two channels for receiving and processing information, words and pictures. CLT then posits the constraints on processing, noting that cognitive load is limited by intrinsic, germane, and extraneous demands on working memory. Ultimately, Richard Mayer extended these theories to posit guidance on how to help learners retain and apply new knowledge using multimedia.

The CTML postulates that using a combination of words and images helps learners attend to relevant information, organize this information into coherent mental representations, and integrate these representations into long-term memory for later retrieval [12–14].

**Fig. 1:**
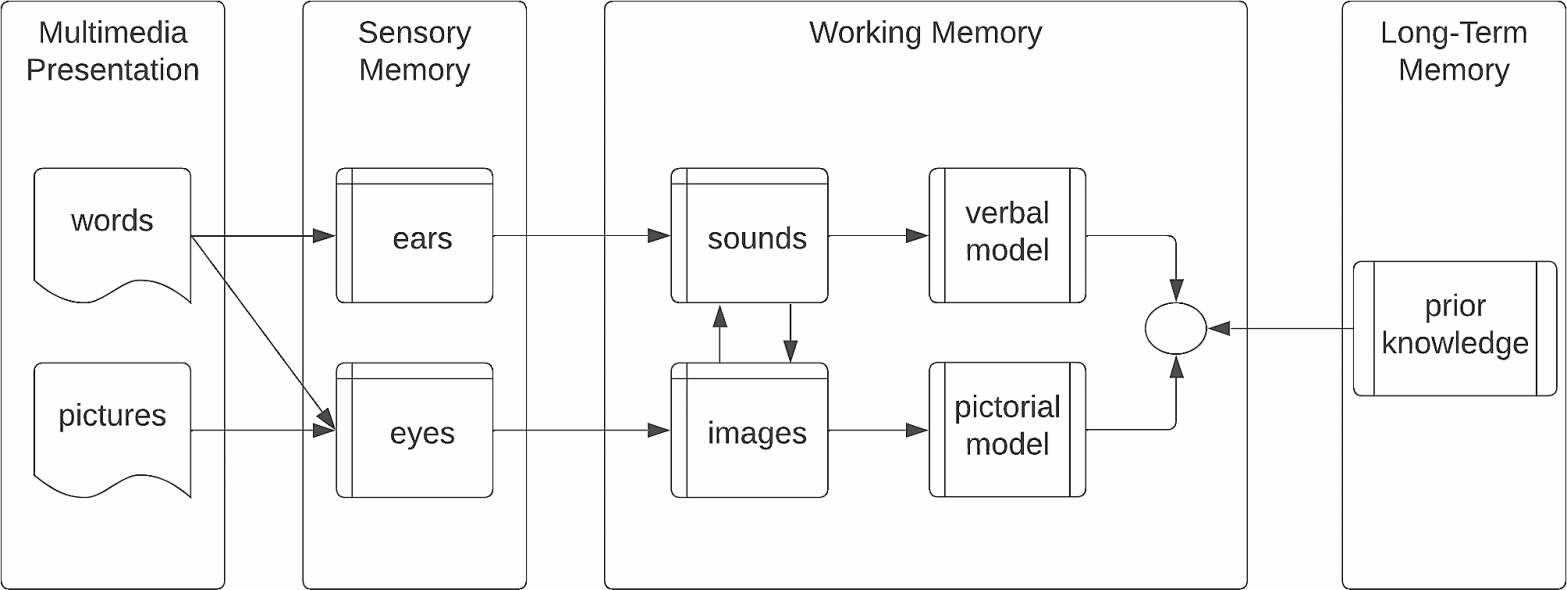
Illustrated depiction of Mayer’s Cognitive theory of multimedia learning

Mayer’s original CTML prescribes six basic principles for using multimedia for learning [4]. First, the multimedia principle concludes that individuals learn better from text combined with content-related images. Instructional materials often apply this principle by using representational pictures to illustrate information; i.e., a diagram depicting the human immune system. Second, spatial contiguity establishes the value of presenting text close to the related images. Third, this same concept applies to other forms of media, such as audio or video files, and is known as the temporal contiguity principle. In other words, separating a media element from its relevant text increases cognitive load unnecessarily, impeding comprehension. Fourth, when learning designs combine narration with images, it applies the modality principle, further enhancing the likelihood of information retention through dual-coding. However, when written text in multimedia duplicates the narration, cognitive overload negates the benefits of media use, otherwise known as the redundancy principle. Lastly, just as decorative images can prove distracting to the learner, extraneous words, pictures, or unnecessary sounds or music can increase cognitive load or cause dissonance. Thus, the coherence principle recommends against such practices.

When applied individually or in combination, attending to the CTML design principles benefits learners cognitively. One study applied CTML surgery clerkship coursework in medical education for Year 3 medical students, finding significantly improved knowledge transfer and retention [15]. A follow-up study in the same program delayed testing to detect applicability for long-term retention, finding significantly higher scores among learners who received the CTML-designed lecture materials [16]. Similar to these findings, an unrelated study found positive student reactions, noting the increased potential for information retention when pharmacy education materials adhered to the principles [17].

The current study uses a similar methodology as the follow-up study [16], testing *PowerPoint* slide designs used with one cohort receiving the traditional design and the second cohort receiving an enhanced design while also having access to the traditional design. Additionally, the current study added the dimensionality of learner motivation to assess student reactions and affective effects of accessing modified media in addition to traditional. In this case, motivation is characterized as interest—the psychological precursor to engage or re-engage [18]. Situational interest, in particular, captures the interaction between an environment and personal preferences, making it suitable as a measure for engagement with multimedia [19]. Situational interest can be further delineated between triggered and maintained [20, 21]. Triggered interest represents the series of emotions and reactions experienced during initial exposure—for example, noticing the colors of a diagram at first glance. Maintained interest represents the deeper value found after prolonged exposure—for example, recognizing the contents of a diagram and finding it a helpful aid. As a theoretical construct, two components comprise maintained situational interest: feeling and value [20]. The feeling-related aspect of maintained situational interest captures a person’s affective engagement, such as enjoyment or appreciation. The value-related aspect of maintained situational interest captures a person’s perception of usefulness. A self-administered survey can assess the situational interest of a learner.

## Methods

The current study examines the impact on student achievement, motivation, and preference using a quasi-experimental design to test redesigning *PowerPoint* slides supporting pharmacology coursework for medical students. The following research questions guided data collection and analysis:

1. What are the achievement differences among medical students who study using slides designed with principles from the *Cognitive Theory of Multimedia Learning*?
2. What are the situational interest differences among medical students who study using slides designed with principles from the *Cognitive Theory of Multimedia Learning*?

### Context

**Fig. 2:**
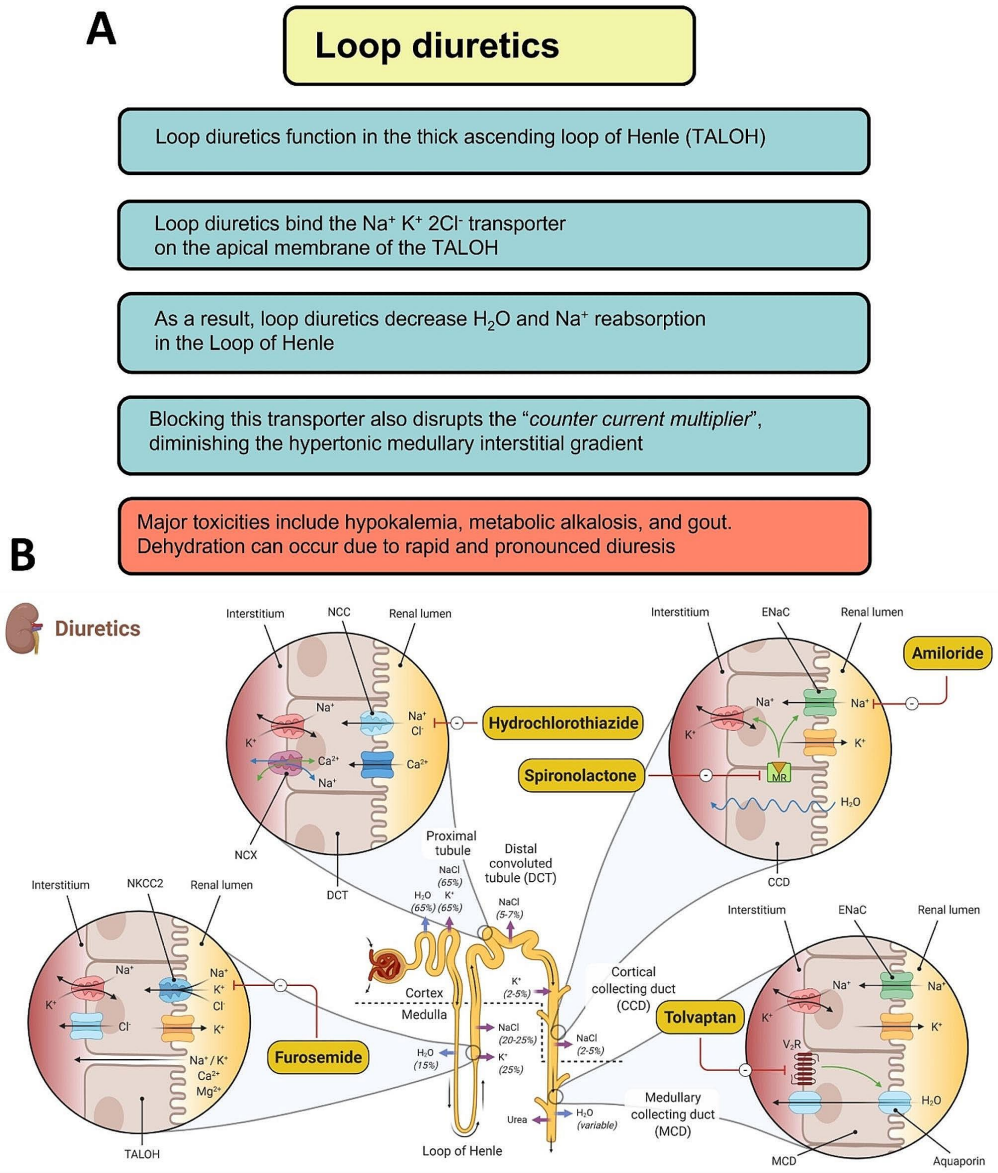
Example slide design. Example original slide in the Cardiac, Pulmonary, and Renal (CPR) block covering the mechanism of action and adverse effects of loop diuretics (**A**). Example redesigned slide representing the mechanism of action of loop diuretics along with the other classes of diuretics covered during the same session (**B**). Loop diuretic adverse effects were covered later in the session on a different slide that also contained a review of the mechanism of action.

The redesigned slides for the current study took into account six critical design decisions. First, the instructor sought to combine all drug content for a single day, including the mechanism of action and physiology/pathology, into a single, high-yield image (Fig. 2B). Second, additional slides with further information, grouping comparisons in the same image or slide (e.g., healthy/disease, disease/disease) and segmenting content into cohesive chunks. Third, the slide redesign emphasized aesthetically pleasing, color-blind friendly illustrations (e.g., using +/- signs to indicate meaning) and colors consistent across the entire curriculum. Fourth, the instructor included worksheet slides, a variation of the redesigned, high-yield images without drug names or labels, and a case study at the end of the lecture to provide application and practice. Fifth, the illustrations relied on visual communication strategies prioritizing symbols and images over text, flow diagrams with colors and symbols, and Venn diagrams to separate the covered drugs into categories. Further, transition slides faded out specific categories to emphasize content about to appear within the context of the broader topic. Sixth, the instructor reused the same slide if the content appeared in multiple places to reinforce the material throughout the curriculum. Figure 2 depicts an original slide (A) and the redesigned slide used with the Experimental group (B).

### Research design

The current study used a convenience sampling strategy, drawing groups from students enrolled in a regional multistate medical education partnership based in the western United States. Specifically, the study focused on the preclinical Foundations Phase of medical education, which encompasses the first 18 months of medical school training and entails the classroom portion of learning. Eight multi-week course blocks cover specific areas of the medical sciences within the Foundations Phase. The blocks include Molecular and Cellular Basis of Disease (MCBD), Invaders and Defenders (I&D), Cardiovascular, Pulmonary, and Renal (CPR), Energy and Homeostasis (E&H), Musculoskeletal (MSK), Blood-Cancer (B&C), Mind-Brain-Behavior (MBB), and Lifecycle (LC). The MSK block did not contain any pharmacology content and was not considered in this study.

Students enrolled at the main campus (*n* = 100) comprised the Traditional group, receiving only the original slides as a mixed-mode of online prerecorded video lectures and in-person lectures as well as *PowerPoint* files. Students enrolled at one of the partner campuses (*n* = 40) constituted the Experimental group, receiving original slides as online prerecorded video lectures and *PowerPoint* files and redesigned slides as in-person lectures and *PowerPoint* files. All students accessed slides via the Canvas learning management system.

Data for the current study included pharmacology block grades (cumulative grades from pharmacology-related exam questions in each block) and cumulative pharmacology grades over the first 18 months of medical education, a self-administered survey completed by 70% of the Experimental group participants (*n* = 28), and system statistics. Individual student grades related to pharmacology exam questions were measured at the end of each block of education and the end of the entire 18 months of education served to establish achievement differences between the groups. The self-administered Situational Interest Survey for Multimedia (SIS-M) is a validated survey instrument (α = 0.9) that measures the primary and secondary constructs of situational interest for research purposes [22]. Statements on the survey include “The *PowerPoint* Slides grabbed my attention” (triggered) and “I am excited about what I learned from the *PowerPoint* Slides” (maintained). Table 1 presents the survey items, with items 1–4 aligning with *SI-triggered* and items 5–12 aligning with *SI-maintained*. Additional questions on the survey asked Experimental group participants which slide deck they preferred and why (open-ended) and demographic data for statistical summary. Researchers consulted learning management system statistics, specifically the number of downloads, to verify statements made in the open-ended survey item.

**Table 1:** SIS items

SIS Type	Survey Item
*SI-triggered*	1. The multimedia presentation was interesting.
*SI-triggered*	2. The multimedia presentation grabbed my attention.
*SI-triggered*	3. The multimedia presentation was often entertaining.
*SI-triggered*	4. The multimedia presentation was so exciting, it was easy to pay attention.
*SI-maintained-feeling*	5. What I learned in the multimedia presentation is fascinating to me.
*SI-maintained-feeling*	6. I am excited about what I learned in the multimedia presentation.
*SI-maintained-feeling*	7. I like what I learned in the multimedia presentation.
*SI-maintained-feeling*	8. I found the information in the multimedia presentation interesting.
*SI-maintained-value*	9. What I studied in the multimedia presentation is useful for me to know.
*SI-maintained-value*	10. The things I studied in the multimedia presentation are important to me.
*SI-maintained-value*	11. What I learned in the multimedia presentation can be applied to my job.
*SI-maintained-value*	12. I learned valuable things in the multimedia presentation.

### Data collection

Students in the Experimental group received a survey invitation by email after the fall 2021 term. A total of *n* = 28 in the Experimental group completed the survey. The survey asked students to consent to participate and respond to the 12-item SIS-M twice, first referencing the original slides and then to the redesigned slides and answering additional preference questions during the second presentation. Student pharmacology grades related to pharmacology exam questions were measured at the end of each block and overall pharmacology grades were measured at the end of the first 18-months of medical education. Grades from all students at each site were used for analysis.

### Data analysis

Researchers used SPSS to analyze the students’ grades and SIS-M survey results. Achievement data was reported as final pharmacology grades separated by block or total combined pharmacology grade over the first 18-months of medical education. An independent sample t-test method was used to evaluate student pharmacology grade differences between all students in the Traditional group (received only original slides) and all students in the Experimental group (received both traditional and redesigned slides). The SIS-M survey analysis accounted for the multiple dimensions of situational interest discussed previously: triggered interest, maintained-value, maintained interest, and maintained-feeling. Given the parametric nature of the data, four paired t-tests were used to evaluate Experimental group students’ interest in the original and redesigned slides. Thematic analysis was performed with ChatGPT (GPT4-Turbo) including generating initial codes and searching for themes [23–27]. From there the researchers discussed the themes to refine overlap and relevancy. This reduced the output to four key themes, eliminating two themes and collapsing two. The themes eliminated did not address specific aspects of slide design. Support for application was collapsed into engagement and renamed to reflect the overlap.

## Results

**Fig. 3:**
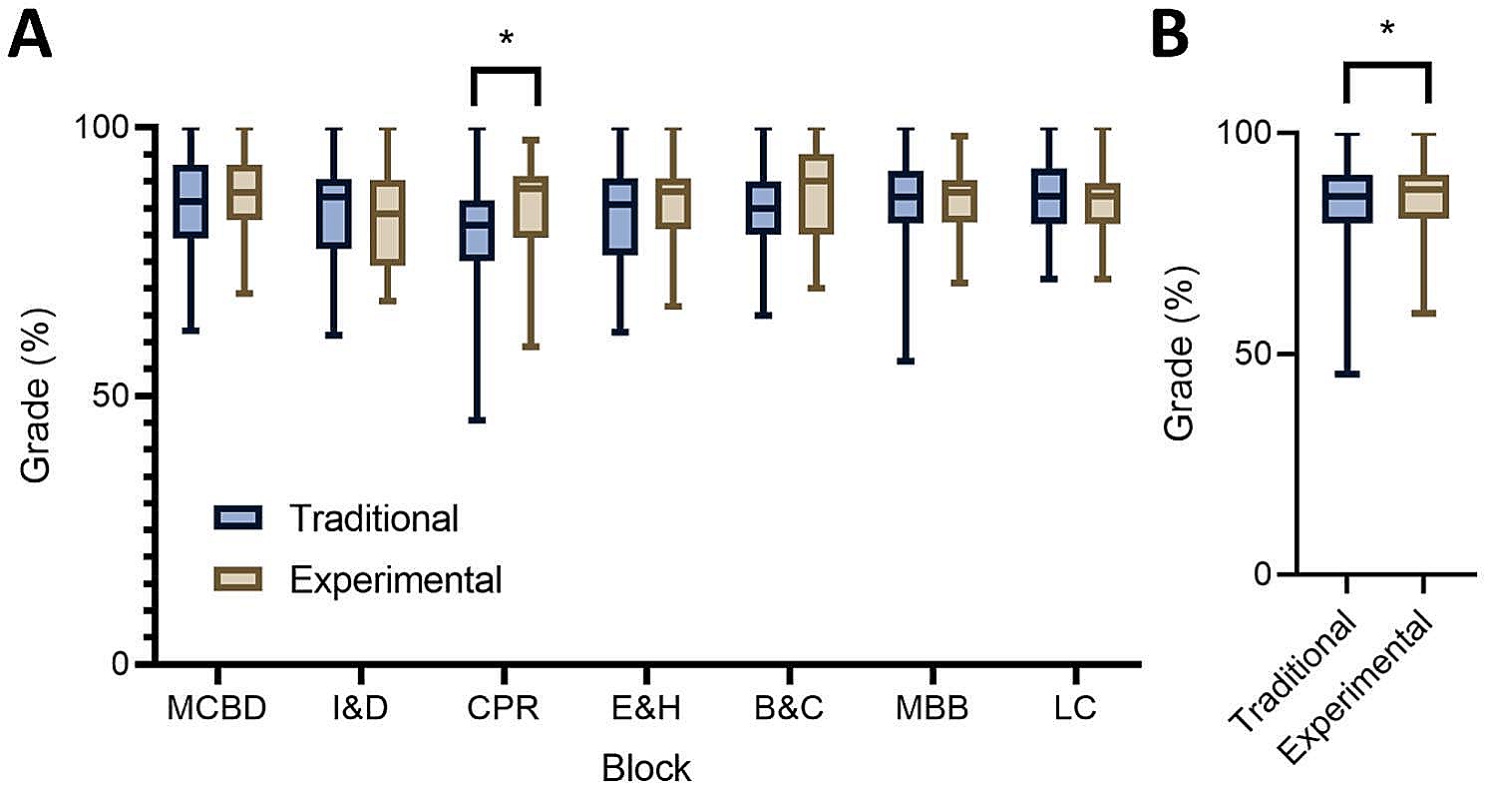
Analysis of student pharmacology performance. Student pharmacology grades related to exam questions segregated by block (**A**). Overall pharmacology grades combined over the entire first 18-months of medical education (**B**). *p < 0.05

Research Question 1 asked: What are the achievement differences among pharmacology students who study using slides designed with principles from the *Cognitive Theory of Multimedia Learning*? We analyzed participants’ pharmacology grades throughout the first 18-months of their coursework to answer this question. Unfortunately, due to the anonymous nature used to collect the SIS-M survey data, we could not determine the grades of students who participated in the survey. Thus, the analysis uses all students’ grades at both sites (*N* = 140, Traditional: *n* = 100, Experimental: *n* = 40). Figure 3 illustrates the results of this analysis.

Through analyzing pharmacology grades by block, results revealed a statistically significant increase in CPR grades in the Experimental group compared to the Traditional group (Traditional: 80.6% ± 0.9, Experimental: 84.8% ± 1.4, *p* = 0.013) (Fig. 3A). All other blocks showed no statistical difference in achievement scores (Fig. 3A). Analysis of pharmacology grades combined throughout the first 18 months of medical education showed a modest but significant increase in the Experimental group compared to the Traditional group (Traditional: 84.7% ± 0.3%, Experimental: 85.9% ± 0.5% *p* = 0.043) (Fig. 3B). Overall, the data suggest that the redesigned slides either increased achievement or had no effect on learning.

Research Question 2 asked: What are the situational interest differences among pharmacology students who study using slides designed with principles from the *Cognitive Theory of Multimedia Learning*? To answer this question, we conducted four paired samples t-tests to examine whether the participants in the Experimental group were more interested in the redesigned slides than the original slides. Table 2 summarizes the four paired sample t-test statistical results. The results indicated a statistically significant difference in participants’ interest between the redesigned slides and original slides, with 100% (*n* = 28) of participants in the Experimental group indicating a preference for the redesigned slides.

**Table 2:** Paired sample T-tests for triggered situational interest (Trig), maintained interest (MT), maintained-feeling (MF), and maintained-value (MV)

	Paired Differences					t	df	Significance	
	Mean	Std. Deviation	Std. Error Mean	95% Confidence Interval of the Difference					
				Lower	Upper			One-Sided p	Two-Sided p
Original Trig– Redesign Trig	-1.62	1.35	0.26	-2.14	-1.09	-6.334	27	< 0.001	< 0.001
Original MT– Redesign MT	− 0.98	0.76	0.14	-1.28	− 0.69	-6.824	27	< 0.001	< 0.001
Original MF– Redesign MF	-1.17	0.90	0.17	-1.51	− 0.82	-6.878	27	< 0.001	< 0.001
Original MV– Redesign MV	− 0.79	0.79	0.15	-1.10	− 0.49	-5.332	27	< 0.001	< 0.001

Participants in the Experimental group indicated a significantly higher average triggered situational interest in redesigned slides (M = 4.85, SD = 0.25) than the original slides (M = 3.23, SD = 1.40), *t*=-6.33, *p* < 0.001.

The results for maintained interest indicated that the students’ interest rating of the redesigned slides (M = 4.87, SD = 0.24) was significantly greater than that of the original slides (M = 3.89, SD = 0.86), *t*=-6.824, *p* < 0.001. The 95% confidence interval for the mean difference between the two ratings was − 1.28 to − 0.69.

The results for maintained**-**feeling (MF) interest indicated that the redesigned slides (M = 4.83, SD = 0.34) interest rating was significantly greater than that of the original slides (M = 3.66, SD = 0.99), *t*=-6.878, *p* < 0.001. The 95% confidence interval for the mean difference between the two ratings was − 1.51 to − 0.82.

The results for maintained-value (MV) interest indicated that the interest rating of the redesigned slides (M = 4.92, SD = 0.19) was significantly greater than that of the original slides (M = 4.13, SD = 0.85), *t*=-5.332, *p* < 0.001. The 95% confidence interval for the mean difference between the two ratings was − 1.10 to − 0.49.

The thematic analysis of the open-ended survey responses in the Experimental group revealed several key themes regarding students’ preferences for pharmacology slide design:

1. Visual Appeal and Organization: Students appreciated slides that were visually appealing, well-organized, and not overwhelming. Comments emphasized the importance of engaging, colorful, and intuitive designs that made studying more interesting and memorable.
2. Use of Diagrams and Visuals: Diagrams and visuals were highlighted as crucial for explaining drug mechanisms and effects. Students found that slides with cartoon-esque images, animations, and clear graphics made content easier to learn and categorize. Visual representations of the material, particularly the visual representation of the drugs’ mechanism of action, were favored over textual descriptions.
3. Consistency and Format: Consistent formatting was a significant theme. Students valued high-yield slides that provided a comprehensive overview of drugs and their mechanisms in one place, making for quick reviews and easier understanding. They appreciated the predictable structure where crucial information was highlighted consistently across slides.
4. Engagement and Application: Engaging content that was easy to follow and memorable was crucial. This included interesting case studies and summary slides that coalesced important details into one place for easier studying. Students appreciated slides that supported application and review, including end-of-lecture questions with mock patient cases and blank review slides for practice. These elements helped solidify understanding and reinforce contraindications.

The thematic analysis indicates a strong preference for slide designs that are visually engaging and well-organized. Students valued consistent formatting, clear and memorable visuals, and elements that supported application and review.

The demographic data from the survey results indicated that 56% of respondents were male and 44% were female, with the majority identifying as Caucasian/white.

## Discussion

In this study, we redesigned *PowerPoint* slides in support of pharmacology content learning to determine the impact of following CTML design principles on the achievement and situational interest of medical students in the preclinical Foundations Phase of medical training. The findings of this study align with similar work in the field, adding additional evidence in favor of using standards-designed media to aid in student achievement. Moreover, the findings indicate a significant difference in learner motivation, measured by the Situational Interest Survey for Multimedia (SIS-M).

### Achievement

The findings indicated that the redesigned slides either supported students’ learning of pharmacology content or had no detrimental effect on their achievement scores. In addition to the overall achievement difference between groups, Experimental group students’ grades in one of the blocks (CPR) increased after using the redesigned slides. The findings are expected given the previous studies in this area and the assumption that using media designed with the CTML principles benefits learning [15, 16].

When asked about their preference, the students shared specific examples of how the redesigned slides assisted their learning. The thematic analysis of first-year medical students’ preferences in pharmacology slide design revealed several crucial insights, highlighting the significance of visual appeal, organization, use of diagrams and visuals, consistency in format, and a strong emphasis on engagement and application in educational material design. Students’ preference for visually appealing and well-organized slides supports previous studies that also found a positive relationship between these aspects of multimedia design and achievement [28–30].

Additionally, the desire for consistent formatting in slides and the appreciation for comprehensive overview slides reflect the students’ need for streamlined content that aids in quick revision and understanding. This is in line with principles of effective instructional design, which emphasize consistency for better information assimilation. Figure 1 depicts how the *Cognitive Theory of Multimedia Learning* perceives the relationship between long-term memory and visio-spatial sketchpad, also known as working memory. Thus, the student’s comment highlights the powerful nature of carefully designing learning media to enhance knowledge transfer.

### Situational interest

The results of the study related to learners’ situational interest suggest increased situational interest in all dimensions when presented with the redesigned slides. However, while all types of situational interest were significantly greater among the Experimental group, the differences in triggered interest and maintained-feeling interest were most noticeable. The sampling of student statements from the open-ended survey items further supports these statistical findings, stating the students’ perceived value of the redesigned slides. Revisiting Fig. 2 for reference, we posit that the richness of the illustrations and application of contiguity principles in the redesigned slides served as a stronger arousal mechanism than the traditional design. This conclusion draws upon previous studies that used eye-tracking methods to correlate increased attention to detailed illustrations [31].

Further, the use of multiple CTML design principles visible in the instructor’s critical design decisions may have contributed to higher maintained-feeling interest. Specifically, using aesthetic, accessible images to support visual communication likely enhanced students’ affective reactions, resulting in greater maintained-feeling situational interest, as illustrated by the student statements, particularly S12. Other studies in this area reveal a strong relationship between aesthetic design and engagement, positing direct influence on enjoyment or liking and the potential to reduce anxiety [32, 33]. Thus, the current findings further extend the research, adding the interest dimensionality to the discussion.

Additionally, these findings highlight the reciprocal relationship between motivation and achievement. Motivation plays a significant role in self-regulation and self-efficacy, and both skills are widely recognized as essential for academic success [34, 35]. Thematic analysis of student responses reveal engagement and application of the material further supporting that CTML design principles, the aesthetic, pleasing design triggered students’ interest. The statements affirm the application of CTML principles around segmenting and organizing content to enhance learning as a form of *maintained-interest-value*. The outcome of this cycle becomes apparent in the participants’ significantly higher achievement and situational interest scores.

### Limitations and confounding variables

While the results strongly support the use of redesigned media to support achievement and motivation among medical students, the current study is not without limitations. The first limitation is how the two groups received the media. The Traditional group learned the material in a mixed-mode through mostly out-off-class video instruction with some in-person lectures, while the Experimental group learned mostly through in-person lectures with the opportunity to receive the original media via out-of-class video instruction. This delivery difference and other related instructional design differences may indirectly influence situational interest.

This second limitation involves Experimental group access to both sets of slides with varying exposure levels, which may have contributed to their higher achievement. Furthermore, the Traditional group students were taught by multiple instructors through the Foundations Phase, while the Experimental group students received instruction from the same instructor. While all students in the Experimental group received an invitation to complete the survey, only 70% responded (*n* = 28) resulting in a small sample size. The quasi-experimental design of the achievement analysis further complicated the study sample, resulting in unequal groups (Experimental = 40; Traditional = 100). Similarly, student privacy protections prevented access to baseline knowledge data; i.e., the researchers’ request for some types of student data were denied during the initial study design. The current resulting findings only support achievement demonstrated within the learning experience without accounting for possible prior knowledge impacts.

Lastly, the study context represents a passive learning environment in which instruction occurs primarily through lecture and content review. Thus far, all implementations of the SIS-M have assessed the motivational impact of multimedia designs in passive learning environments as encountered online or in large-scale settings. Applying the instrument or otherwise replicating the study in an active learning environment will add valuable context to the discussion. Overall, we do not believe that these limitations hinder the study findings.

## Conclusion

Despite endorsement by the Association of American Medical Colleges Institute for Improving Medical Education, using the principles to intentionally design media in medical education to target knowledge transfer has not permeated practice [17]. Thus, the current study adds to the small but growing area of interest to better support medical student success. Additionally, the current study adds to a growing body of evidence that multimedia design is equally important to learner motivation as it is cognition.

Finally, we note that distributed models of medical education with main and partner sites create the novel opportunity to implement design-based research studies with quasi-experimental methods. The multiple locations often create enrollment-capped groups in relatively equal size to aid confidence in data analysis. Additionally, the embedded nature of learning problems in design-based research methods presents a convenient context in which to conduct educational research with purpose [36, 37].

## Data Availability

The datasets generated and/or analyzed during the current study are not publicly available due to institutional policy on studies using student data, but are available from the corresponding author on reasonable request. High-yield slides used in the study are freely available at www.blandpharm.com.

## Abbreviations

CTML: Cognitive Theory of Multimedia Learning
SIS-M: Situational Interest Survey for Multimedia
IPT: Information-Processing Theory
DCT: Dual-Coding Theory
CLT: Cognitive Load Theory
